# Adjuvant therapy with toceranib for hepatocellular carcinoma and cholangiocarcinoma in a Pomeranian

**DOI:** 10.17221/49/2022-VETMED

**Published:** 2023-02-23

**Authors:** Lee Choi, Jin-Young Choi, Hun-Young Yoon, Kieun Bae, Kyong-Ah Yoon, Jung-Hyun Kim

**Affiliations:** ^1^Department of Veterinary Internal Medicine, College of Veterinary Medicine, Konkuk University, Gwangjin-gu, Seoul, Republic of Korea; ^2^Department of Veterinary Surgery, College of Veterinary Medicine, Konkuk University, Gwangjin-gu, Seoul, Republic of Korea; ^3^Department of Veterinary Biochemistry, College of Veterinary Medicine, Konkuk University, Gwangjin-gu, Seoul, Republic of Korea

**Keywords:** anticancer effect, biliary carcinoma, canine

## Abstract

A 10-year-old spayed female Pomeranian dog was referred for hepatic mass evaluation. Blood tests revealed mildly elevated alkaline phosphatase activities. Computed tomography revealed a mass with multiple nodules on the right hepatic medial lobe adjacent to the caudal *vena cava*; histopathology confirmed mixed hepatocellular carcinoma (HCC) and cholangiocarcinoma (CC). Because of incomplete resection, adjuvant therapy was recommended. As tumour cells showed *PDGFR*-α, *c-Kit*, and *FGFR1* overexpression, the anticancer effect of tyrosine kinase inhibitors was evaluated on the cells; toceranib was the most effective and was administered starting with an extra-labelled dose. The dog remained stable for 2.3 years with mild adverse effects. To our knowledge, this is the first successful clinical application of toceranib in a dog with mixed HCC-CC.

Hepatocellular carcinoma (HCC) and cholangiocarcinoma (CC) represent the most and second most common primary hepatic malignant tumours in dogs, accounting for up to 77% and 9–41% of hepatic tumours, respectively (Patnaik et al. 1980; Hammer and Sikkema 1995; Cullen and Popp 2002). However, mixed hepatocellular cholangiocarcinoma (mixed HCC-CC) is a rare form of primary malignant hepatic tumour that exhibits histologically differentiated features of both HCC and CC. The incidence of mixed HCC-CC is variably reported between 0.4% and 14.2% among primary hepatic tumours in humans (Maximin et al. 2014; Stavraka et al. 2018). However, reports on the incidence of mixed HCC-CC in small animals are limited. Mixed HCC-CC displays aggressive clinical behaviour and is associated with a more favourable prognosis than CC but poorer prognosis than HCC (Stavraka et al. 2018). The overall survival and recurrence rates of mixed HCC-CC after hepatic lobectomy or transplantation are affected by the percentage of the CC component in the mixed tumour; a high CC component is associated with poor survival (Wu et al. 2016).

Receptor tyrosine kinases (RTK) mediate cell growth, proliferation, angiogenesis, and tissue repair, and are expressed on the cell membrane. Although their activation is finely controlled in normal cells, RTK dysregulation caused by mutations can lead to uncontrolled proliferation, aberrant survival, and neovascularisation. This abnormal signalling leads to the development of neoplasms (Vail et al. 2019). Tyrosine kinase inhibitors (TKI) are small-molecule inhibitors that block the downstream signalling of RTK (Vail et al. 2019). Toceranib phosphate is a TKI commonly used in veterinary oncology and is a targeted agent approved by the Food and Drug Administration (Vail et al. 2019). It can target c-Kit, vascular endothelial growth factor receptor (VEGFR) 2, platelet-derived growth factor receptor β (PDGFR-β), and FMS-like tyrosine kinase 3 (FLT3) and thus inhibits multiple targets (Vail et al. 2019). Toceranib exerts therapeutic effects against both canine mast cell tumours and variant solid tumours, including anal sac adenocarcinoma, gastrointestinal stromal tumour, thyroid carcinoma, and nasal adenocarcinoma (Vail et al. 2019). Herein, we describe the clinical outcome of toceranib therapy for treating an incompletely excised mixed HCC-CC in a dog.

## Case presentation

**Figure 1:**
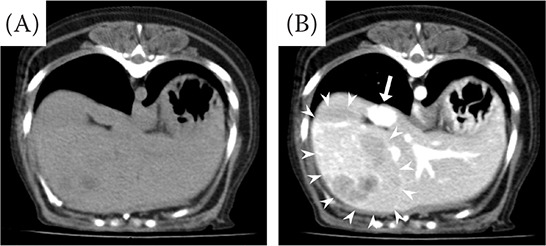
Computed tomography of the dog diagnosed with mixed HCC-CC (A) Plain and (B) postcontrast transverse view of the abdomen showing a heterogeneous hyperattenuating mass with heterogeneous hypoattenuating multiple nodules on the right medial lobe (arrowheads) adjacent to the caudal *vena cava* (arrow) HCC-CC = hepatocellular carcinoma-cholangiocarcinoma

**Figure 2:**
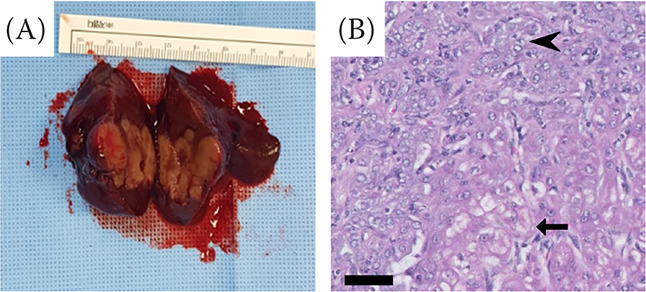
Gross morphological and histopathological features of hepatic mass in a dog with mixed HCC-CC (A) Note the multiple nodules within the massive hepatic tumour. (B) Histopathology of hepatic mass revealed that it was composed of both neoplastic hepatocytes and the biliary epithelium. Neoplastic hepatocytes contain abundant vacuolated cytoplasm and irregular oval nuclei with small nucleoli (arrow). The neoplastic biliary epithelium is cuboidal or polygonal with eosinophilic cytoplasm and irregular oval nuclei with variably prominent nucleoli (arrowhead). Haematoxylin & eosin (H&E) stain; magnification 400 ×; scale bar = 50 μm HCC-CC = hepatocellular carcinoma-cholangiocarcinoma

A 10-year-old spayed female Pomeranian dog was referred for evaluation of a hepatic mass discovered during a medical check-up. Complete blood count and serum chemistry analyses showed no remarkable findings, except a mildly elevated alkaline phosphatase level of 3.66 μkat/l (reference interval 0.38–3.54 μkat/l). Abdominal ultrasonography (US) and computed tomography (CT) revealed a 47.0 × 31.2 × 40.7-mm massive, irregular, and heterogenous mass on the right hepatic medial lobe adjacent to the caudal *vena cava* (Figure 1). The mass exhibited multifocal hyperechoic nodules, some of which had several hypoechoic cavitary lesions. The thorax and adjacent abdominal lymph nodes were unremarkable. Right medial liver lobectomy was performed following cholecystectomy using a thoracoabdominal stapling device with three rows of staples (Figure 2A). Tissue samples were subjected to histopathologic evaluation, confirming that the mass represented a mixed HCC-CC with incomplete excision margins (Figure 2B).

**Figure 3:**
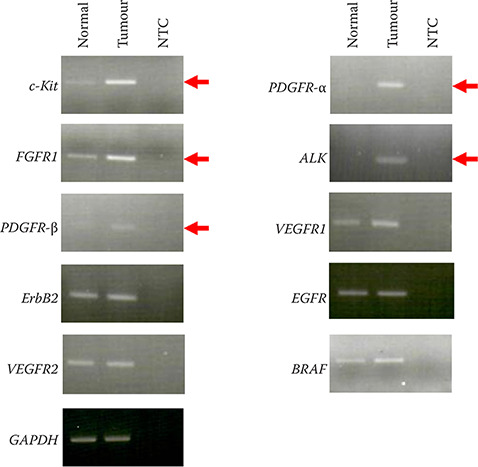
mRNA expression of RTK in normal and tumour samples from a dog with mixed HCC-CC Note that *PDGFR*-α, *c-Kit*, and *FGFR1* in tumour cells were predominantly overexpressed compared with those in normal hepatocytes. *GAPDH* was used as the housekeeping gene *ALK* = anaplastic lymphoma receptor tyrosine kinase; *BRAF* = B-Raf proto-oncogene serine/threonine-protein kinase; *c-KIT* = stem cell factor receptor; *EGFR* = epidermal growth factor receptor; *ErbB2* = erb-b2 receptor tyrosine kinase; *FGFR* = fibroblast growth factor receptor; *GAPDH* = glyceraldehyde-3-phosphate dehydrogenase; HCC-CC = hepatocellular carcinoma-cholangiocarcinoma; NTC = non-template control; *PDGFR* = platelet-derived growth factor receptor; *RTK* = receptor tyrosine kinase; *VEGFR* = vascular endothelial growth factor receptor

**Figure 4:**
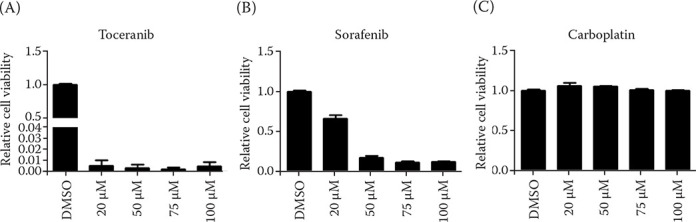
*In vitro* chemosensitivity of tumour cells from a dog with mixed HCC-CC Toceranib (A), sorafenib (B), and carboplatin (C) were applied for an *in vitro* chemosensitivity test. Compared with that of sorafenib and carboplatin, toceranib exhibited more potent antitumor activity at the lowest concentration of 20 μM. In contrast, carboplatin did not induce cell death even at a high concentration of 100 μM DMSO = dimethyl sulfoxide; HCC-CC = hepatocellular carcinoma-cholangiocarcinoma

Adjuvant chemotherapy was recommended to treat the residual tumour tissues. Targeted therapy was chosen as the first treatment option, considering the side effects on normal cells and hepatocytic drug resistance. To determine the appropriate targeted therapeutic agent, an RTK gene expression analysis and *in vitro* chemosensitivity test were performed. The mRNA expression levels of RTK were compared between the resected tumour tissue and adjacent normal tissues through reverse transcription-polymerase chain reaction. *PDGFR-*α, *PDGFR-*β, *c-Kit*, *FGFR1*, and anaplastic lymphoma receptor tyrosine kinase (*ALK*) were overexpressed in the tumour tissues compared with their levels in normal tissues (Figure 3). Based on RTK gene expression analysis data [electronic supplementary material (ESM) Figure S1], toceranib and sorafenib, which can block these RTK, as well as carboplatin, were selected. Next, their cytotoxic effects on cells isolated from the resected tumour tissues were evaluated via an *in vitro* chemosensitivity test (Figure 4). The cytotoxic effect of sorafenib treatment increased in a dose-dependent manner. The relative cell viabilities after treatment with 20 μM toceranib and sorafenib were < 1% and 68%, respectively. As 20 μM toceranib treatment induced significant cell death, its concentration was lowered and the effects were analysed (see ESM Figure S2).

Additionally, carboplatin did not induce a cellular response at a high concentration of 100 μM, indicating that the tumour cells were chemoresis-tant. Toceranib was therefore considered the most suitable drug in this case.

Toceranib (Palladia^®^, Zoetis, Florham Park, NJ, USA) administration was started at 2.35 mg/kg per os (p.o.) every other day (e.o.d.), which was within the extra-labelled dose range (2.2–2.7 mg/kg p.o. e.o.d.). The dose was adjusted based on the degree of adverse effects and the dog’s quality of life. When possible, the drug was maintained at the previously mentioned dose without interruption. The lowest dose prescribed was 1.52 mg/kg. Furthermore, the hepatic protectants silymarin (Legalon^®^; Bukwang Pharm., Seoul, Republic of Korea; 5 mg/kg orally twice per day) and ursodeoxycholic acid (Ursa^®^; Daewoong Pharm., Seoul, Republic of Korea; 7.5 mg/kg orally twice per day) were administered concurrently.

A medical recheck for adverse effects of the drug and the possibility of tumour recurrence and metastasis was performed approximately once per week for the first 6 weeks and then every 6–8 weeks. All adverse effects were graded according to the Veterinary Cooperative Oncology Group’s common terminology criteria for adverse effects (VCOG-CTCAE v1.1). Although the dog tolerated the toceranib treatment well, adverse effects such as mild anorexia (grade 1 of 4), mild to moderate neutropenia (grade 1–2 of 4), and hypopigmentation of the nose were observed. Neutropenia severity induced by the bone marrow-suppressive effects of toceranib was dose-dependent. This cytotoxic effect was observed for toceranib dosages > 2.43 mg/kg on average. Following a drug dose of approximately 25% reduction, the neutrophil count normalised within one week. However, mild anorexia (grade 1 of 4) persisted. To maintain the appetite of the dog, the client received dietetic consultation and coaxed the dog with frequent dietary changes.

Tumour recurrence and metastasis were monitored by measuring the levels of serum liver enzymes and α-fetoprotein as well as through abdominal US and CT scans. Following liver lobectomy, liver enzyme levels normalised within one month, and no remarkable changes associated with toceranib treatment were observed. As α-fetoprotein levels were not measured before surgery, they could not be compared before and after surgery. However, after surgery, the α-fetoprotein level was maintained at 0.9 ng/ml (reference interval < 70 ng/ml). No significant findings were observed on the abdominal US performed every three months on average, and no metastasis or recurrence was observed by performing a CT scan one year after diagnosis. Despite a plan to perform CT annually, a CT scan two years after diagnosis was put on hold concerns about the risk of anaesthesia due to congestive heart failure diagnosed in periodic checkups. However, fortunately, no remarkable changes were found in the US evaluation during the follow-up. The dog remained stable for 833 days (approximately 2.3 years) without moderate or severe systemic complications induced by the anticancer therapy.

## DISCUSSION AND CONCLUSIONS

Conventional chemotherapy exerts broad adverse effects because chemotherapeutic agents can damage rapidly dividing normal cells (Vail et al. 2019). Hepatocytes exhibit remarkable chemoresistance because of the P-glycoprotein expressed on their cell membrane and the ability of these cells to efflux drugs (Vail et al. 2019). Furthermore, hepatic tumours show poor therapeutic responses to conventional chemotherapy, with a chemotherapy response rate of < 20% in humans (Kim et al. 2017; Vail et al. 2019). In veterinary oncology, encouraging results were not obtained in a study that used gemcitabine and carboplatin to treat dogs with unresectable HCC (Dominguez et al. 2009).

Neoplasms including HCC are caused by the overexpression or mutation of genes encoding proteins associated with growth and proliferation signalling (Becker et al. 2007; Llovet and Bruix 2008; Moeini et al. 2012); thus, targeted anticancer agents that block these proteins have attracted the attention of clinicians because of their potential as safer alternatives to chemotherapeutic agents in humans (Seebacher et al. 2019). Therefore, we hypothesised that targeted therapy would be more appropriate to reduce the adverse effects and to have a better anticancer effect than conventional chemotherapy.

Our results for RTK gene expression analysis revealed that the patient overexpressed *PDGFR*-α, *PDGFR*-β, *c-Kit, FGFR1*, and *ALK*. Toceranib and sorafenib were the TKI candidates chosen for targeting PDGFR and c-Kit, and an *in vitro* chemosensitivity test was performed. Although sorafenib, a multi-kinase inhibitor that targets Raf-1, B-Raf, VEGFR 2, PDGFR, and c-Kit receptors, is the only FDA-approved targeted anticancer agent for human liver cancer (Lang 2008; Llovet and Bruix 2008), we found that toceranib was superior to sorafenib in inducing tumour cell death. Therefore, toceranib was used to treat this patient.

Although the label dose of toceranib is 3.25 mg/kg e.o.d., a previous study reported that lower doses have clinical benefits because of reduced adverse effects (Bernabe et al. 2013). To achieve low toxicity and fewer drug holidays, orally administered toceranib was prescribed to the patient at concentrations of 1.52–2.67 mg/kg e.o.d. Because the manufacturer stated that the provided tablets should not be ground or split, fine adjustment of the drug dose challenged for a patient with a weight of about 3 kg.

The most common adverse effects associated with toceranib phosphate in canine patients include constitutional symptoms (anorexia and weight loss), gastrointestinal symptoms (diarrhoea and gastrointestinal bleeding), bone marrow suppression (neutropenia and anaemia), musculoskeletal problems (lameness), renal toxicity (azotaemia and proteinuria), hepatic toxicity (elevated level of liver enzymes), and dermatologic complications (depigmentation) (Bernabe et al. 2013). Neutropenia was the most worrisome adverse effect observed in this patient after toceranib administration. Targeted agents such as sorafenib and imatinib can inhibit the FLT3 and c-kit signal pathways, thereby inhibiting haematopoiesis. Therefore, toceranib, which can block c-Kit, FLT3, may have caused bone marrow suppression by inhibiting RTK involved in haematopoiesis (Gilliland and Griffin 2002; Barber et al. 2011). According to the VCOG-CTCAE criteria, dose reduction of the anticancer drug is not indicated in grade 1 and grade 2 neutropenia (LeBlanc et al. 2021). However, to maintain the patient’s immunity and prevent infections, the dose of toceranib was reduced by approximately 25% on average when mild to moderate neutropenia was observed. Following the reduction of the toceranib dose, the neutrophil count was restored to the normal range. Our strategy helped the patient continue the anticancer treatment with reduced toxicity and fewer drug holidays.

Anorexia, one of the adverse effects observed in the patient, was affected by the duration of administration rather than the dose of toceranib. Despite the temporary reduction in toceranib dose to alleviate anorexic symptoms, the appetite rarely normalised. Anorexia caused weight loss, which increased the patient’s intolerance to the anticancer drugs. Thus, restoring appetite was important for the quality of life of the patient and the client.

The nose hypopigmentation observed in this patient was considered drug-induced depigmentation. A previous case report described skin depigmentation in a dog treated with toceranib; however, this discoloration improved within several weeks after the discontinuation of toceranib administration (Cavalcanti et al. 2017).

Our patient has been living in relatively good health without severe adverse effects, recurrence, or metastasis for more than two years since incomplete surgical resection and toceranib treatment. However, the lack of metastasis may be also partly explained by the low rate of metastasis in HCC tumours. As the median overall survival is longer than two years even for incomplete excision procedures, massive HCC has a good prognosis (Matsuyama et al. 2017; Vail et al. 2019). Alternatively, massive CC has a poor prognosis because of its aggressive behaviour. Following surgical excision, the mean survival time of patients is approximately six months because of local recurrence and metastasis (Vail et al. 2019). Little is known about the prognosis of mixed HCC-CC because of the rarity of this type in veterinary medicine. A recent retrospective study reported that the prognosis of HCC-CC with only surgical treatment was similar to that of HCC. However, this outcome has statistical limitations due to the small sample size to suggest a treatment plan and prognosis for incompletely resected HCC-CC (Terai et al. 2022). A human study indicated that the median survival time of mixed HCC-CC is between that of massive HCC and massive CC (Stavraka et al. 2018).

To our knowledge, this is the first report of the successful use of toceranib for long-term management of incompletely resected mixed HCC-CC in a Pomeranian dog. This case report provides valuable information on the efficacy of toceranib therapy and the prognosis of mixed HCC-CC, which is rare in veterinary medicine. Veterinarians should carefully consider toceranib as an adjuvant therapy option for treating dogs with incompletely resected mixed HCC-CC.

## Electronic Supplementary Material

Supplementary Figures
