# Supplementary material for: Adjuvant therapy with toceranib for hepatocellular carcinoma and cholangiocarcinoma in a Pomeranian
Source: Vet Med (Praha). 2023 Feb 23;68(2):83–9. doi: 10.17221/49/2022-VETMED (PMC10847815; doi:10.17221/49/2022-VETMED)
Supplement: Supplementary Figures [file VETMED-68-122049-s001.pdf]

https://doi.org/10.17221/49/2022-VETMED

Adjuvant therapy with toceranib for hepatocellular carcinoma and cholangiocarcinoma in a Pomeranian

*Lee Choi[1], Jin-Young Choi[2], Hun-Young Yoon[2], Kieun Bae[3], Kyong-Ah Yoon[3], Jung-Hyun Kim[1]**

[1]*Department of Veterinary Internal Medicine, College of Veterinary Medicine, Konkuk University, Gwangjin-gu, Seoul, Republic of Korea*
[2]*Department of Veterinary Surgery, College of Veterinary Medicine, Konkuk University, Gwangjin-gu, Seoul, Republic of Korea*
[3]*Department of Veterinary Biochemistry, College of Veterinary Medicine, Konkuk University, Gwangjin-gu, Seoul, Republic of Korea*

**Corresponding author: junghyun@konkuk.ac.kr*

The authors are fully responsible for both the content and the formal aspects of the electronic supplementary material. No editorial adjustments were made.

Electronic Supplementary Material (ESM)

Figure S1. RT-PCR for comparison of RTK gene expression

Figure S2. Tendency of cell death for toceranib concentration

https://doi.org/10.17221/49/2022-VETMED

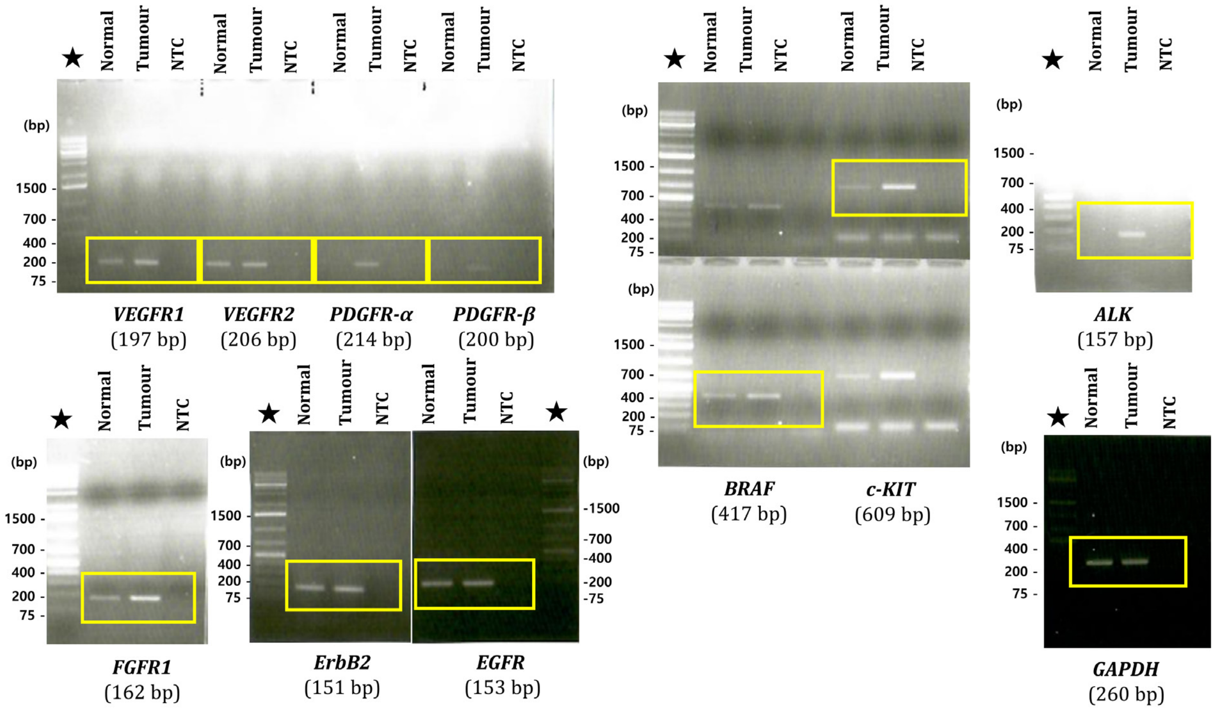

Figure S1. RT-PCR for comparison of RTK gene expression

RT-PCR was conducted to compare the mRNA expression levels of RTK between the tumour tissues and adjacent normal tissues from a dog with mixed HCC-CC. *PDGFR*-α, *PDGFR*-β, *c-Kit*, *FGFR1*, and *ALK* in tumour cells were overexpressed compared to those in normal hepatocytes; in particular, the overexpression of *PDGFR*-α, *c-Kit*, and *FGFR1* was predominant. Yellow boxes indicate the band of normal hepatocytes, tumour cells, and NTC in order. Star symbols indicate DNA size markers.

ALK = anaplastic lymphoma receptor tyrosine kinase; *BRAF* = B-Raf proto-oncogene serine/threonine-protein kinase; *c-KIT* = stem cell factor receptor; DNA = deoxyribonucleic acid; *EGFR* = epidermal growth factor receptor; *ErbB2* = erb-b2 receptor tyrosine kinase; *FGFR* = fibroblast growth factor receptor; *GAPDH* = glyceraldehyde-3-phosphate dehydrogenase; HCC-CC = hepatocellular carcinoma-cholangiocarcinoma; NTC = non-template control; *PDGFR* = platelet-derived growth factor receptor; RTK = receptor tyrosine kinase; RT-PCR = reverse transcription-polymerase chain reaction; *VEGFR* = vascular endothelial growth factor receptor

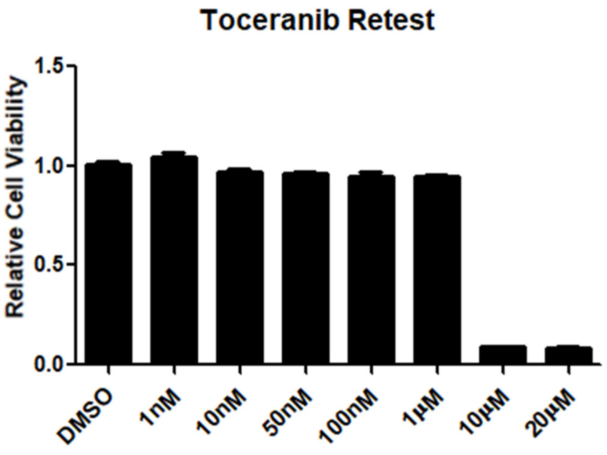

Figure S2. Tendency of cell death for toceranib concentration

Because toceranib induced significant cell death at 20 μM, an in vitro chemosensitivity test was also performed at lower concentrations. The level of cell death at 10 μM was as high as that at 20 μM.

DMSO = dimethyl sulfoxide
